# Comment on: ‘Comparison of GPT-3.5, GPT-4, and human user performance on a practice ophthalmology written examination’ and ‘ChatGPT in ophthalmology: the dawn of a new era?’

**DOI:** 10.1038/s41433-023-02773-9

**Published:** 2023-09-28

**Authors:** Nima Ghadiri

**Affiliations:** 1 Department of Ophthalmology, Liverpool University Hospitals NHS Foundation Trust, Liverpool, UK; 2 Department of Eye and Vision Science, University of Liverpool, Liverpool, UK

**Keywords:** Technology, Education

I read with great interest two recent articles promulgating the use of large language models (LLMs), specifically ChatGPT, in Ophthalmology.

The first paper by Lin et al. [1] compared the performance of two LLMs - GPT-3.5 and GPT-4 - to human ophthalmologists on a 260-question ophthalmology exam. They found GPT-4 and humans performed similarly overall, both exceeding the passing threshold, while GPT-3.5 did not pass. However, both LLMs struggled with image-based and higher-order questions compared to text-based, fact recall questions.

The second paper by Ting et al. [2] delves into the broader potential of ChatGPT in the medical field, including Ophthalmology. The authors outline the platform’s architecture and training methodology and highlight the potential across patients, professionals, research, and education, including self-diagnosis, generating patient education materials, assisting clinical decision making, and enhancing medical training. However, they note limitations like inaccuracy, outdated information, lack of transparency, and potential biases.

While the rapid advances of ChatGPT and other LLMs are laudable and hold potential throughout ophthalmology and medicine, it is vital to approach their implementation with caution. As shown by Lin et al., difficulties with visual interpretation and complex reasoning remain. And as Ting et al. discuss, without transparency and accountability, patient harm could result from erroneous information, such as responses which, though plausible, are not accurate. Medical decisions should be substantiated by evidence and reliable sources, and a lack of transparency and explainability raises ethical concerns.

Therefore, rather than relying solely on ChatGPT, which is a proprietary black box, I believe the field would also benefit from openly developed LLMs that prioritize transparency, ethics, and partnerships with ophthalmologists. It is also essential to consider the suitability of particular AI language models for specific medical specialties. For instance, while GPT-4 demonstrated promising results in ophthalmology, it struggled with imaging-based questions. This limitation indicates that a one-size-fits-all approach may not be appropriate for every medical domain.

Presently, there exists an abundance of LLMS, which exist at various stages of development and implementation within healthcare [3]. Initiatives such as Anthropic’s Constitutional AI [4], aligned with human values, offer an alternative approach so that we can cautiously embrace, rather than uncritically adopt, this exciting technology. AI language models which are specifically designed and trained for medical applications can even complement the capabilities of general-purpose LLMs.

In conclusion, while LLMs like GPT-3.5 and GPT-4 have shown promise in medical applications, it is crucial to avoid over-reliance on a single model and to consider specialized alternatives. Integrating diverse AI models and continually refining their capabilities will pave the way for responsible and effective adoption of AI in healthcare. We urge researchers, healthcare professionals, and stakeholders to carefully assess the strengths and limitations of different AI models, to ensure safe and accurate medical practices.
